# IGF-1 and Chondroitinase ABC Augment Nerve Regeneration after Vascularized Composite Limb Allotransplantation

**DOI:** 10.1371/journal.pone.0156149

**Published:** 2016-06-07

**Authors:** Nataliya V. Kostereva, Yong Wang, Derek R. Fletcher, Jignesh V. Unadkat, Jonas T. Schnider, Chiaki Komatsu, Yang Yang, Donna B. Stolz, Michael R. Davis, Jan A. Plock, Vijay S. Gorantla

**Affiliations:** 1 Department of Plastic Surgery, Thomas E. Starzl Transplant Institute, University of Pittsburgh, Pittsburgh, Pennsylvania, United States of America; 2 McGowan Institute for Regenerative Medicine, University of Pittsburgh, Pittsburgh, Pennsylvania, United States of America; 3 Department of Trauma Surgery, East Hospital of Shanghai, Shanghai, China; 4 Center for Biological Imaging, University of Pittsburgh, Pittsburgh, Pennsylvania, United States of America; 5 United States Army Institute for Surgical Research, San Antonio Military Medical Center, 3698 Chambers Road, San Antonio, Texas, United States of America; University of Sydney, AUSTRALIA

## Abstract

Impaired nerve regeneration and inadequate recovery of motor and sensory function following peripheral nerve repair remain the most significant hurdles to optimal functional and quality of life outcomes in vascularized tissue allotransplantation (VCA). Neurotherapeutics such as Insulin-like Growth Factor-1 (IGF-1) and chondroitinase ABC (CH) have shown promise in augmenting or accelerating nerve regeneration in experimental models and may have potential in VCA. The aim of this study was to evaluate the efficacy of low dose IGF-1, CH or their combination (IGF-1+CH) on nerve regeneration following VCA. We used an allogeneic rat hind limb VCA model maintained on low-dose FK506 (tacrolimus) therapy to prevent rejection. Experimental animals received neurotherapeutics administered intra-operatively as multiple intraneural injections. The IGF-1 and IGF-1+CH groups received daily IGF-1 (intramuscular and intraneural injections). Histomorphometry and immunohistochemistry were used to evaluate outcomes at five weeks. Overall, compared to controls, all experimental groups showed improvements in nerve and muscle (gastrocnemius) histomorphometry. The IGF-1 group demonstrated superior distal regeneration as confirmed by Schwann cell (SC) immunohistochemistry as well as some degree of extrafascicular regeneration. IGF-1 and CH effectively promote nerve regeneration after VCA as confirmed by histomorphometric and immunohistochemical outcomes.

## Introduction

Vascularized composite allotransplantation (VCA) is the new frontier of organ transplantation that has enabled restorative options for devastating civilian or combat craniofacial or extremity injuries. The focus of most ongoing research in VCA is to develop novel immunomodulatory or tolerogenic therapies to minimize or eliminate life-long immunosuppression in VCA, which are predominantly life-enhancing procedures. Nevertheless, the life long burden of immunosuppression is not the only hurdle to VCA [1]. Neuroregeneration remains the other major, yet relatively understudied barrier in VCA. Functional outcomes after clinical VCA such as upper extremity or face transplantations have been acceptable in some patients, but in many cases, motor and even more so sensory function, have remained unsatisfactory. Slow or sub-optimal nerve regeneration or delayed muscle re-innervation (or denervation), could all predispose to poor recovery. Such inadequate or ineffective nerve regeneration can result in loss of graft function, which equates to graft “failure” even in an immunologically viable graft. This is a feature unique to VCA in contrast to solid organs. Hence, for broader feasibility of VCA, it is imperative that we explore strategies that facilitate rapid and effective nerve regeneration as well as timely distal target reinnervation. The goal of this study was to test the neurotherapeutic efficacy and feasibility of Insulin-like Growth Factor-1 (IGF-1) and/or chondroitinase ABC (CH) in VCA. These agents have been well studied in models of peripheral nerve grafts, and nerve crush, transection and repair [2–14]. Our earlier study on the individual efficacy of CH, was the first to demonstrate significant improvements in nerve morphometry in a stringent allogeneic rat limb VCA [14]. In the current study, we hypothesized that IGF-1 and CH may have additive, complementary or synergistic benefit in enhancing morphological and immunohistological outcomes of axonal regeneration after nerve transection and repair in VCA.

## Materials and Methods

### Animals and Surgical Procedures

Eight- to 12-week-old male Lewis (recipients) and Brown Norway rats (donors) [Harlan Laboratories (Indianapolis, IN) (250–300g)] were used in fully mismatched limb transplantation. Animals were anesthetized with intraperitoneal (IP) pentobarbital (40mg/kg injection) or isoflurane gaseous anesthesia (V-1 Lab, VetEquip Inc., CA, USA). Orthotopic hind-limb transplantation was performed as described earlier [14]. Donor and recipient hind limbs were shaved and retrieved through a circumferential skin incision, ligation of epigastric vessels, microdissection of femoral vessels, and transection at the level of the inguinal ligament. The donor leg was amputated at mid-femoral level with transection of the femoral vessels proximally to provide length for anastomoses. The femoral artery was flushed with 5ml of cold heparinized Ringer’s lactate solution and stored at 4°C. The recipient leg was amputated similarly but with transection of the vessels more distally than the donor. Osteosynthesis was performed using an 18-gauge needle for intramedullary fixation. The sciatic nerve was coaptated using 9–0 nylon sutures (Microsurgery Instruments, Inc., Bellaire, TX) and the thigh muscles were sutured with interrupted 4–0 Vicryl sutures (Ethicon Inc., Somerville, NJ). Microsurgical anastomosis of the femoral artery was performed with interrupted 11–0 nylon sutures. The femoral vein was coapted using a cuff technique with a polyamide tube used as a microcuff (RiverTech Medical, Chattanooga, TN).

An overdose of pentobarbital (80 mg/kg) or CO2 exposure was used for euthanasia and all efforts were made to minimize pain and distress. This study was carried out in strict accordance with the University of Pittsburgh Institutional Animal Care and Use Committee and American Association for Laboratory Animal Care guidelines.

### Experimental Design

Rat recipients were allocated into five groups (n = 5). Experimental groups, dosing, route and frequency of administration of treatments are shown in Table 1. Daily IGF-1 injections were avoided in the CH group to eliminate any confounding effects of IGF-1. All animals received FK506 (0.5mg/kg daily, IP) as baseline immunosuppression for five weeks (end point of experiment) to prevent acute VCA rejection. We chose five weeks as our end point, as there is a “blow-through” effect observed after nerve repair around 40 days post-surgery, whereby an advancing nerve front overcomes an experimental nerve defect. This effect renders nerve regeneration differences between experimental and control groups indistinguishable at late time points (further details in Discussion).

**Table 1:** Experimental Groups, Dosing, Administration, Frequency of Treatment.

Treatment (n = 5)	Dose	Site of Administration	Timing
Control (PBS)	30 mL	Distal nerve; sub-epineurial	Prior to transection
IGF-1	200ng/30mL	Distal nerve; sub-epineurial	Prior to transection
	200ng/30mL (IGF-1)	IM	After limb transplant
CH	1U/30uL	Distal nerve; sub-epineurial	Prior to transection
IGF-1 + CH	200ng/30mL (IGF-1)	Distal nerve; sub-epineurial	Prior to transection
	1U/30mL (CH)	Distal nerve; sub-epineurial	Prior to transection
	200ng/30mL (IGF-1)	IM	After limb transplant

PBS, phosphate buffered saline; IGF, Insulin-like growth factor-1 (Recombinant, R&D Systems, MN); CH, chondroitinase ABC (Sigma-Aldrich, MO)

The Control (PBS) and CH groups received individually administered doses as three sub epineurial injections (10μL each) along distal nerve prior to transection.

The IGF-1 group received three sub-epineurial injections (10μL each) along distal nerve prior to transection followed by daily intramuscular injections (200ng/30mL) into transplanted limb.

The IGF-1+CH group received three sub-epineurial injections of IGF-1 mixed with CH (30μL total volume) along distal nerve prior to transection followed by daily intramuscular injections of IGF-1 (200ng/30mL) into transplanted limb.

Gastrocnemius muscles and sciatic nerves from hind limbs (operated and naïve) were harvested at endpoint for histomorphometric and immunohistochemical (IHC) analyses. The total length of the rat sciatic nerve prior to branching (trifurcation) into deep muscular branches is approximately 2 cm (20 mm). We performed coaptation of the sciatic nerve at the middle point. To ensure that samples were collected proximal to the branching of the sciatic nerve, we chose sites at 5 and 10 mm distal to the coaptation for histomorphometry and immunostaining, respectively. We collected two samples per nerve, one fixed in glutaraldehyde (for histomorphometry) and the other for IHC and immunofluorescent staining (S1 Fig). Quantitative histomorphometry of sciatic nerves was performed on sections fixed in glutaraldehyde. Given the limitation of available nerve tissues for sampling, some immunostaining of sciatic nerves was done on prior resin embedded samples (after resin stripping) while other samples were derived from cryosections after fixation in Bouin’s fixative.

### Quantitative Histomorphometry

#### Gastrocnemius muscle

Explanted hind limbs were fixed in 2% paraformaldehyde for 24 hours. Bones were decalcified using CalRite (Thermo Scientific, MA, USA) and mid limb sections (10–20 mm thick) were embedded in paraffin. Five μm muscle sections were cut and stained using Mallory’s Trichrome method (Rowley Biochemical Institute, MA, USA) per manufacturer recommendations. Cross-sectional images were evaluated blindly using brightfield microscopy and MetaMorph Microscopy Automation and Image Analysis software (Version 7.7.5.0). Single line and trace region tools were used to measure perimeter of randomly selected smallest and largest muscle fibers and area of muscle fascicle, respectively. When using the line tool, we chose only well-rounded muscle fibers (S2A Fig). Measured parameters also included number of fibers per fascicular area; mean area of a fiber calculated from the total area of fascicle divided by the number of fibers and mean percent of connective tissue. The images were thresholded for blue color (connective tissue) and this area was expressed as a percent with references to total tissue.

#### Sciatic nerve

Sciatic nerves (2 cm long samples, including the coaptation site) were collected from explanted limbs at euthanasia, and carefully mounted on wooden tongue depressors. For histomorphometry, a 10 mm long nerve segment containing the coaptation site in the middle (see S1 Fig) was cut and fixed in a cold 2.5% glutaraldehyde solution. Nerve samples (2.5 mm long distal segment) were excised after 24 hours of fixation, post-fixed with 1% osmium tetroxide, dehydrated in ethanol, embedded in Polybed 812 resin (Polysciences, Warrington, PA, USA), cut into 300 nm thick nerve sections and stained with 1% toluidine blue for light microscopy. Digital microscopy images were obtained and nerve parameters were measured with MetaMorph software. For each nerve (n = 5), a blinded observer analyzed six fields chosen by systematic random sampling at 100x magnification [15]. Parameters included fiber density (per 1 mm^2^), total number of fibers per distal area of nerve, myelin area, axon area, and G-ratio (axon area/total fiber area). Trace region tool was used to measure nerve fiber and axon area, from which myelin area was calculated by subtracting axon area from fiber area (see S2B Fig). When necessary, myelin area was measured manually using the same tool. Only myelinated nerve fibers inside the nerve cross section were counted and analyzed. In all graphs, naive nerve data are shown for reference only.

### Immunohistochemistry and Immunofluorescence Staining

#### Gastrocnemius muscle

Samples were fixed in 4% paraformaldehyde and paraffin-embedded and cut into 5 μm sections. After deparaffinization, rehydration, antigen retrieval with citrate buffer (pH 6.0) for 30 minutes at 98°C, and blocking with normal serum, anti-pAktSer^473^ antibody (1:200) (Millipore, CA, USA) was applied overnight. After 24 hours, secondary biotin-bound antibody (goat anti-mouse, 1:800) was applied for 30 minutes at room temperature (RT). To amplify and then detect positive staining, we used the Vectastain ABC kit and ImmPACT DAB Peroxidase Substrate kit, respectively (both from Vector Laboratories, CA, USA). The sections were counterstained with hematoxylin and after dehydration, cover slipped with Permount. A negative control (without first antibody) was included in each set of experiments and negligible staining was confirmed. Staining was quantified by counting the total number of positively labeled cells/nuclei and data presented as the percentage of positively stained nuclei to the total number of cells per field.

#### Sciatic nerve

For immunohistochemistry and immunostaining, we used two different samples of the nerve: collected 5 mm and 10 mm distal respectively, to the site of coaptation (S1 Fig). Following nerve histomorphometry as described above, samples (5 mm segment distal to the coaptation) were further processed for evaluation of glycosaminoglycan (GAG) content using anti-chondroitin 4-sulphate antibody (Abcam, MA, USA), to assess the effect of CH or IGF-1 (in the proximity of administration) on proteoglycan accumulation after nerve injury. Prior to GAG staining, resin-embedded nerve sections were treated with saturated sodium hydroxide for 20 min to strip off the resin from sections, washed, processed for antigen retrieval and treated with 10% hydrogen peroxide for 20 min to remove the osmium tetroxide. The primary antibody (1:50) was applied overnight with next day staining procedures as described for anti-pAktSer473 antibody staining for gastrocnemius muscle.

Nerve segments (10 mm distal to coaptation) (S1 Fig) were fixed in Bouin’s fixative for 24 hours, washed in phosphate-buffered saline (PBS) with lithium carbonate, and incubated in 5% gelatin + 5% sucrose (PBS) overnight. Samples were cryopreserved in the gelatin+sucrose solution and 10 μm thick sections were cut on a cryotome. Before staining, sections were washed in 37°C PBS, incubated in 0.1M acetic acid solution (37°C) to eliminate gelatin residues, washed again (every step for 10 minutes). Next, the sections were blocked with normal serum and incubated with first antibody (anti-GFAP (1:100, Millipore, CA, USA), anti-S100 (1:50, Abcam, MA, USA), anti-Krox20 (1:50, Covance, NJ, USA), anti-Oct6 (1:50, Santa Cruz Biotechnology, CA, USA), anti-neurofilament medium chain (NF-M) (1:400) and anti-GAP43 (1:500) (both from ThermoScientific/Pierce, IL, USA) overnight at 4°C. Sections with NF-M staining were counterstained with FluoroMyelin according to the manufacturer’s instructions (Life Technologies, Carlsbad, CA, USA). For Oct6, antigen retrieval with pH 6.0 citrate buffer was applied under the same conditions as mentioned for the muscle sections. Respective secondary antibodies were added after washing at the dilution 1:400 or 1:800 for 30 minutes at RT. All samples were counterstained with Hoechst 33258 to visualize nuclei. A negative control was included in every experiment. Staining was examined using NIS Elements software by measuring binary area (area of positive staining) and mean intensity per field. The product of staining was calculated by multiplying binary area by mean intensity.

All data for histomorphometry, IHC and immunostaining were collected from five-six fields per rat (20x, n = 5 per group). Background fluorescence was subtracted as follows from all analyzed images: an image was sampled on ImageJ in a ratio and then degraded by a structuring element; then the degraded image was subtracted from the initial image (i.e. "top hat" transform). In all graphs, naive muscle data are shown for reference only.

### Confocal Imaging

Samples were imaged with Olympus Fluoview 1000 confocal microscope using a 60x oil objective. Immunohistochemistry images were represented as z projections of confocal stacks acquired from serial laser scanning. Images of overall myelin expression were acquired with a Nikon 90i upright microscope using a 20x objective and modified using NIS-Elements software to show only red channel (myelin expression). Muscle images were acquired with a Nikon brightfield microscope.

### Statistical Analysis

All data were imported into Prism 5.0 software (GraphPad Software Inc, CA, USA). Data from sections containing more than one subsample were analyzed for their intra-sample heterogeneity. The subsamples within one section were compared and tested for normality with Kolmogorov-Smirnov test and for heterogeneity of variance with the method of Bartlett, with a significance level of 5%. The data showed normal distribution and heterogeneity was not significant. We used a one-way analysis of variance (ANOVA) as our study was mostly focused on one independent variable with >2 conditions or treatments. If the ANOVA was significant (at least one significant difference between conditions) we performed a Tukey post-hoc test to compare two conditions at a time and determine all possible pair-wise differences (if at least one difference was significantly different from 0). Differences were considered significant for p < 0.05 and were marked with asterisk as follows: * p < 0.05, ** p < 0.01, *** p < 0.001. Data are expressed as mean +/- standard error of the mean (mean+/-SEM., error bars) and the reported ‘n’ values are the numbers of different individual rats from which data were obtained.

## Results

### Nerve Histomorphometry

**Fig 1:**
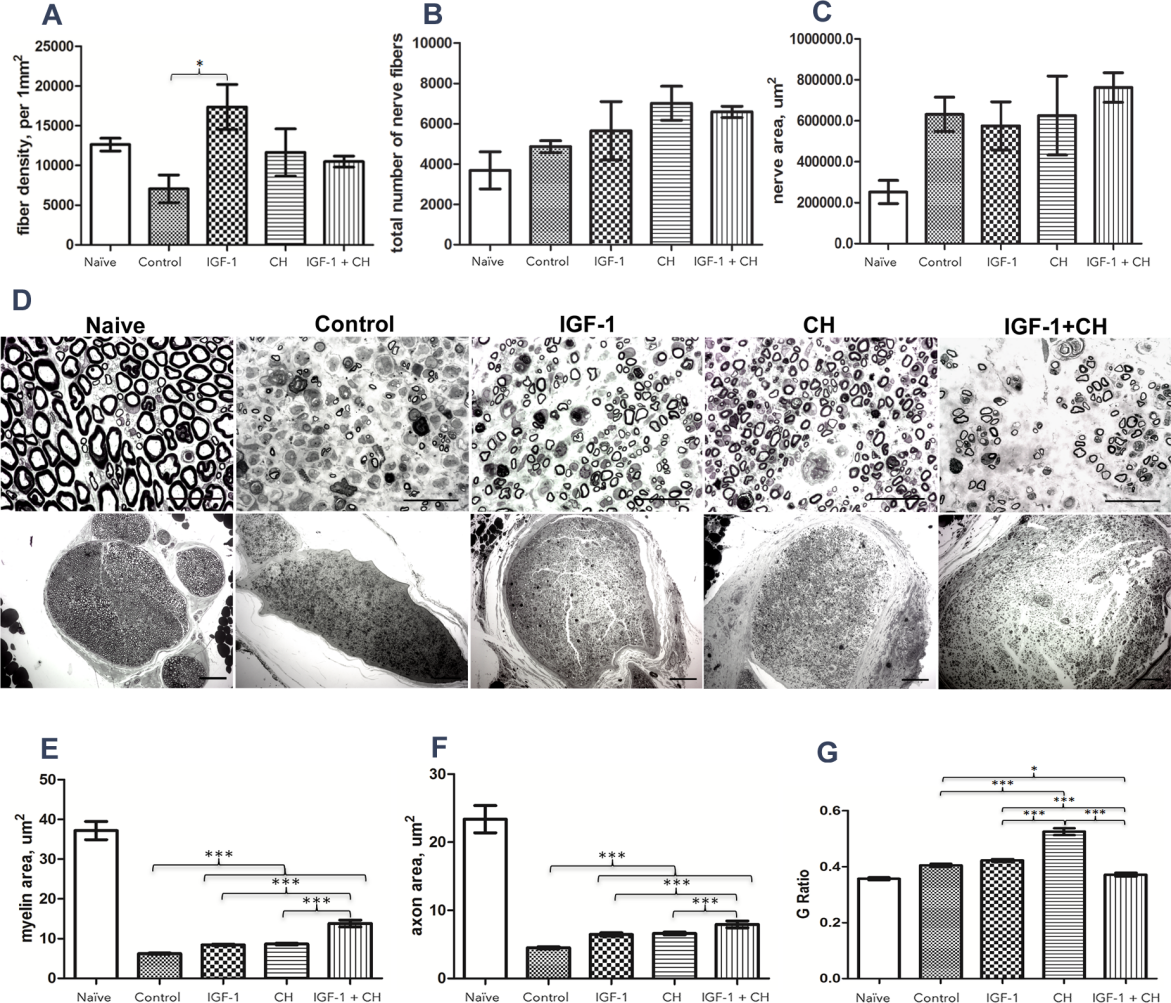
Histomorphometric characterization of nerve regeneration. (A) density of fibers in distal part of the nerve; (B) total number of fibers per distal nerve area (manual count); (C) nerve cross-section area; (D) cross-section samples of distal nerves, 100x (upper raw, scale 10 μm) and 10x (lower raw, scale 50 μm); (E) myelin area; (F) axon area; (G) G-Ratio of nerve fibers. This is the ratio of the axonal area to the total fiber area and is widely utilized as a functional and structural index of optimal axonal myelination (S2B Fig).

Quantitative histomorphometric analysis of nerves is shown in Fig 1. Fiber density in the distal part of the nerve (approximately 5 mm from coaptation) was higher in IGF-1 [mean +/- SEM: 17364+/-2831(p = 0.0243)] as compared to controls. CH (mean +/- SEM: 11649+/-2970) and IGF-1+CH (mean +/- SEM: 10492+/-689) treatment did not result in marked improvement in fiber density. (Fig 1A). Respectively, the total numbers of fibers per nerve area were 1.16, 1.44 and 1.28 fold higher for the IGF-1, CH, and IGF-1+CH groups as compared to controls (Fig 1B). Transplanted nerves demonstrated reduction in fiber size across groups after surgery (Fig 1D). There were also changes in the size of the nerve cross-section as demonstrated by Fig 1D, mid panel. Nevertheless, myelin and axon area increased by 1.35 and 1.43 fold (mean +/- SEM: 8.417+/-0.1819 and 6.459+/-0.2425, p<0.001 in both) and 1.39 and 1.47 fold (mean +/- SEM: 8.658+/-0.2163 and 6.603+/-0.1979, p<0.001 in both) in the IGF-1 and CH groups versus controls (mean +/- SEM: 6.248+/-0.1495 and 4.506+/-0.1471), respectively. Highest increases (mean +/- SEM: 13.81+/-0.859 and 7.939+/-0.5055) (1.76- and 2.2 fold) were seen in the IGF-1+CH groups (Fig 1E and 1F). The G-ratio of was most improved with CH treatment (Fig 1G).

### Nerve Immunohistochemistry

**Fig 2:**
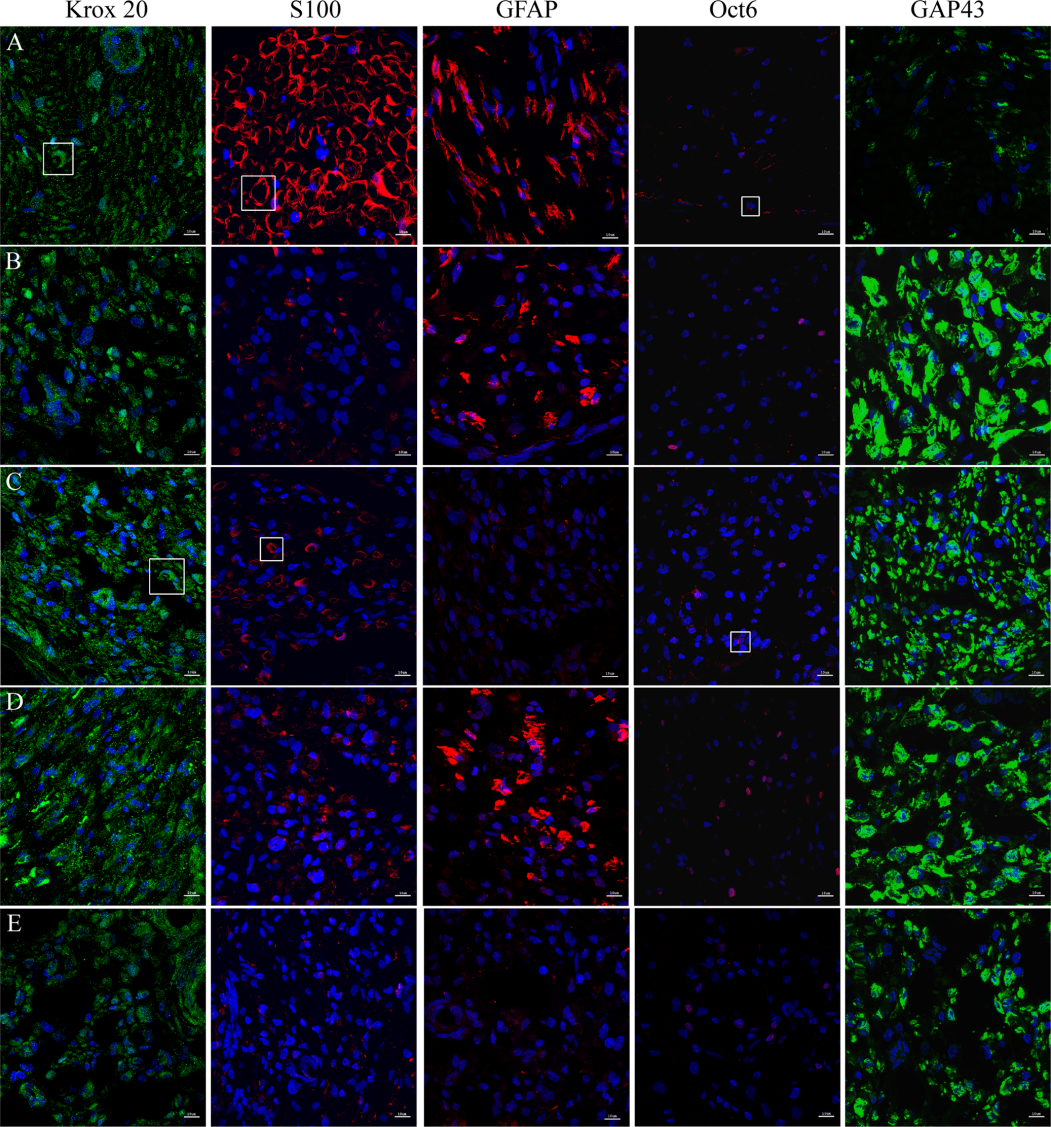
Immunohistochemical staining of the distal nerve. Regeneration marker—GAP43 (green), immature non-myelinating Schwann cells (SCs)—GFAP (red), pre-myelinating SCs—Oct6 (red), committed non-myelinating and myelinating SCs—S100 (red), myelinating SCs—Krox20 (green) are shown. Comparison of expression to that in naïve nerves is indicated in the white boxes. The name of antigen is shown in each column, identification for naive sample and groups is shown in rows: (A row) naive; (B) control; (C) IGF-1; (D) CH; (E) IGF-1+Ch. Magnification 60x, scale 10 μm.

Multiple markers were used to establish the comprehensive temporal or dynamic aspects of nerve regeneration within distal nerve segments. IHC with markers for progression of nerve regeneration such as growth-associated protein (GAP43), axonal growth (Neurofilament M (NF-M)), and characterization of SC populations (S100, GFAP, Oct6, Krox20, myelin) is shown in Fig 2. GAP43 expression was prominent in controls, with lower intensity in IGF-1 and IGF-1+CH groups (as assessed on Tukey, p<0.5, n = 5).

**Fig 3:**
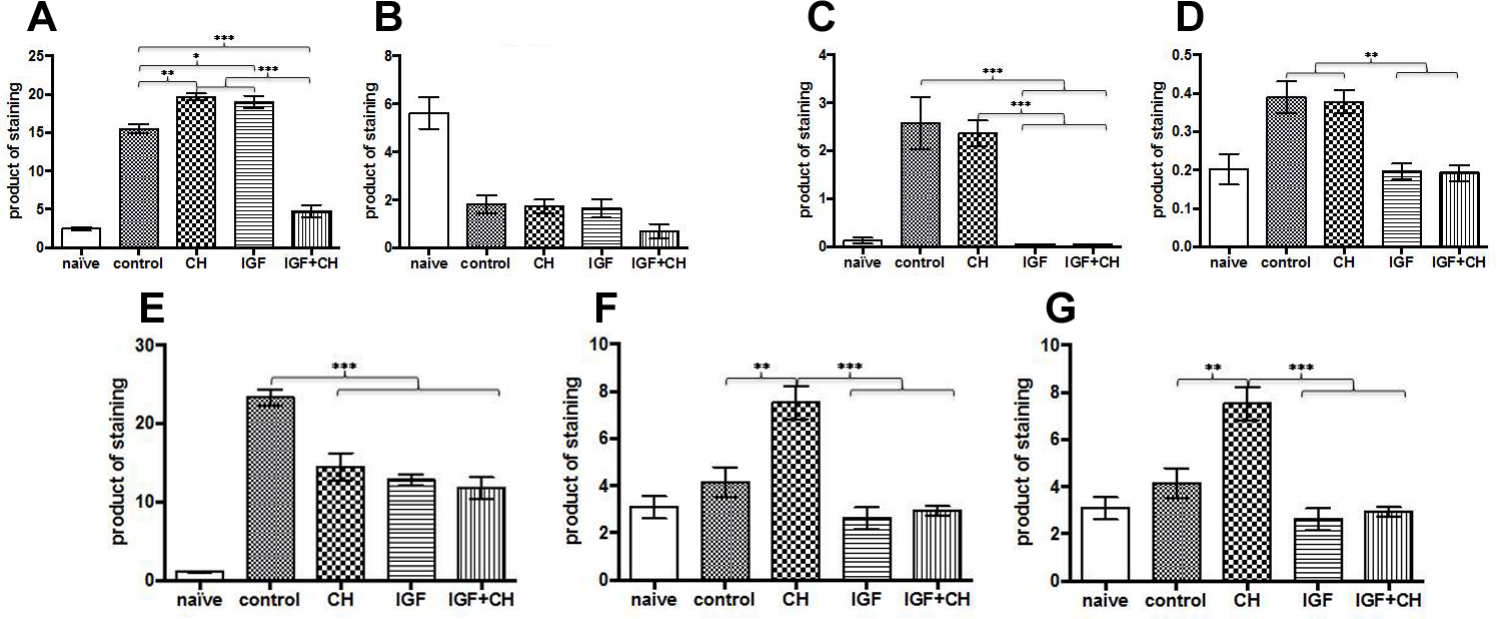
Quantitative representation of differentiation and proliferation markers. Histograms show product of staining of differentiation markers/commited Schwann cells: (A) Krox20, (B) S100; proliferation markers/immature Schwann cells: (C) GFAP, (D) Oct-6; nerve regeneration: (E) GAP43; axonal growth/myelination: (F) NF-M, (G) myelin.

When analyzing samples for SC markers, we focused our evaluation on changes in proliferation (anti-GFAP, Oct6 antibodies) and differentiation (anti-S100, Krox20). GFAP was highly expressed in the CH group with very low expression in IGF-1 and IGF-1+CH samples (as assessed on Tukey’s, p<0.05, n = 5). Similarly, Oct6 expression was rarely observed in the SC nuclei in the IGF-1 and IGF-1+CH samples as compared to the CH group. Instead, its expression was found in some cells in close proximity to the nuclei that resembled its distribution in naïve nerve (see box inserts). S100 expression was significantly decreased across groups (as assessed on Tukey’s, p<0.05, n = 5); but SCs in the CH, IGF-1 and control samples regained on average 26% of the expression seen in naïve nerves. Moreover, some areas of the IGF-1 treated nerve sections had S100 expression similar to those seen in the naïve nerves (see box inserts, “semilunar or donut” shaped areas). Staining for Krox20 was significantly higher in the IGF-1 and CH nerves versus controls (as assessed on Tukey’s, p<0.05, n = 5). In some instances, its expression in IGF-1 samples was similar to naïve nerves (box inserts). These trends were not observed in the remaining groups. The IGF-1+CH group had the lowest expressions of both S100 and Krox20. Fig 3 demonstrates the levels of expression accessed by product of staining (binary area X mean intensity) for all markers, including NFM and myelin.

**Fig 4:**
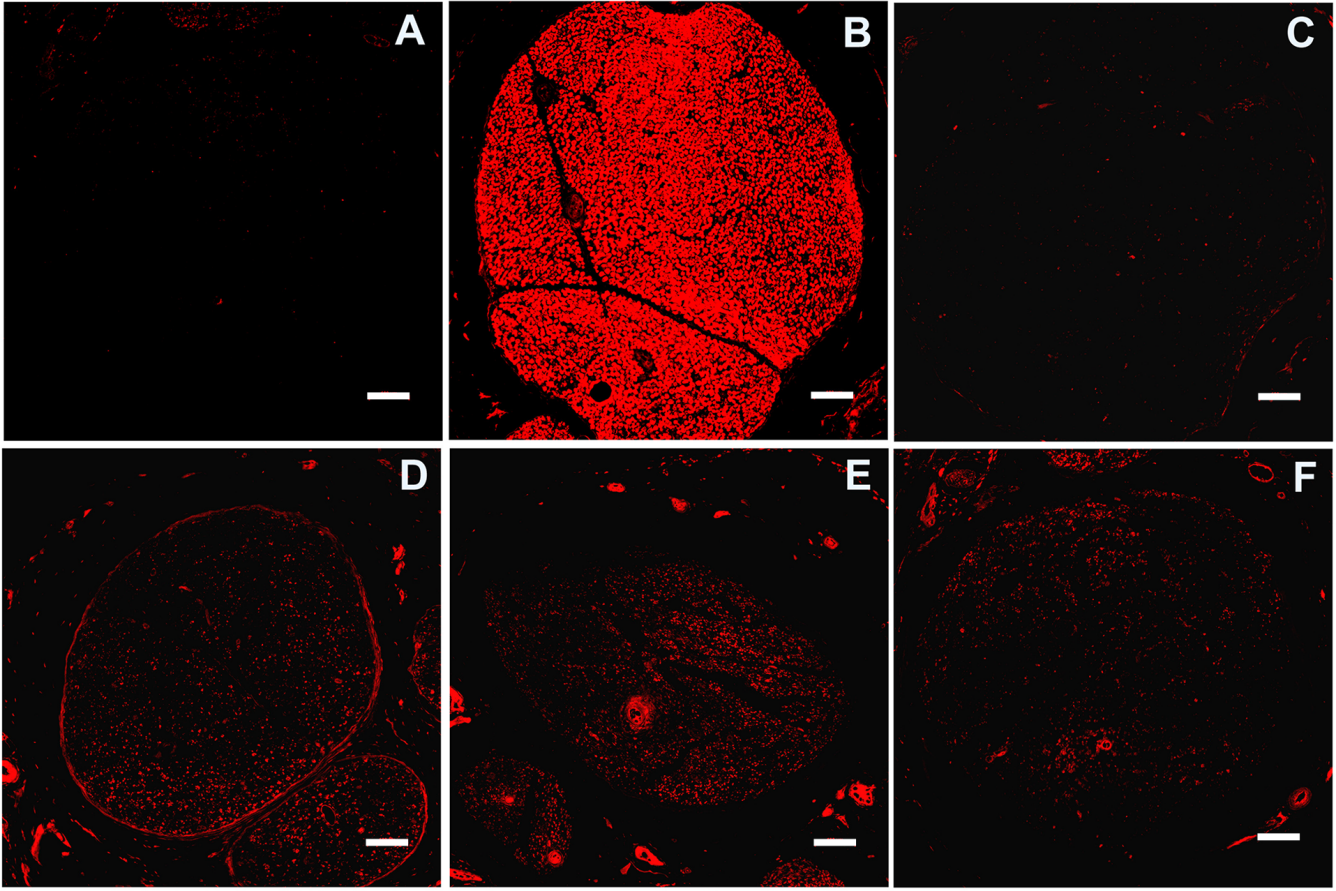
Overall myelin expression. Magnification 20x, scale 100 μm, sections 10 mm distal to surgical coaptation site, 5 weeks post-surgery. Images: (A) negative control (B) naive nerve; (C) control nerve; (D) CH; (E) IGF-1; (F) IGF-1+CH. Red—myelin, blue—nucleus.

We compared overall myelin expression in nerve cross-sections obtained 10 mm distal to the coaptation site (Figs 3 and 4A–4F). We found that overall myelination increased 119, 29, and 36-fold (mean +/- SEM: 7.4+/-0.5843, 1.781+/-0.1732 and 2.249+/-0.1998, p<0.001 all) in the IGF-1, CH, and IGF-1+CH samples versus control (mean +/- SEM: 0.0624+/-0.007), respectively. Fig 4A–4F demonstrates examples of myelin expression in relation to axonal regrowth in groups. Despite the substantial decrease in the expression across groups after surgery, the product of NF-M staining was 1.8 fold higher (mean +/- SEM: 7.511+/-0.6974, p = 0.002) in the CH group whereas in IGF-1 and IGF-1+CH nerve, the staining was 1.6- and 1.4 fold less (mean +/- SEM: 2.624+/-0.4533 and 2.925+/-0.2124) as compared to controls (mean +/- SEM: 4.165+/-0.6294), respectively. Overall, the complex staining demonstrates advanced regeneration in the treatment groups with superior differentiation and regeneration in the IGF-1 group.

**Fig 5:**
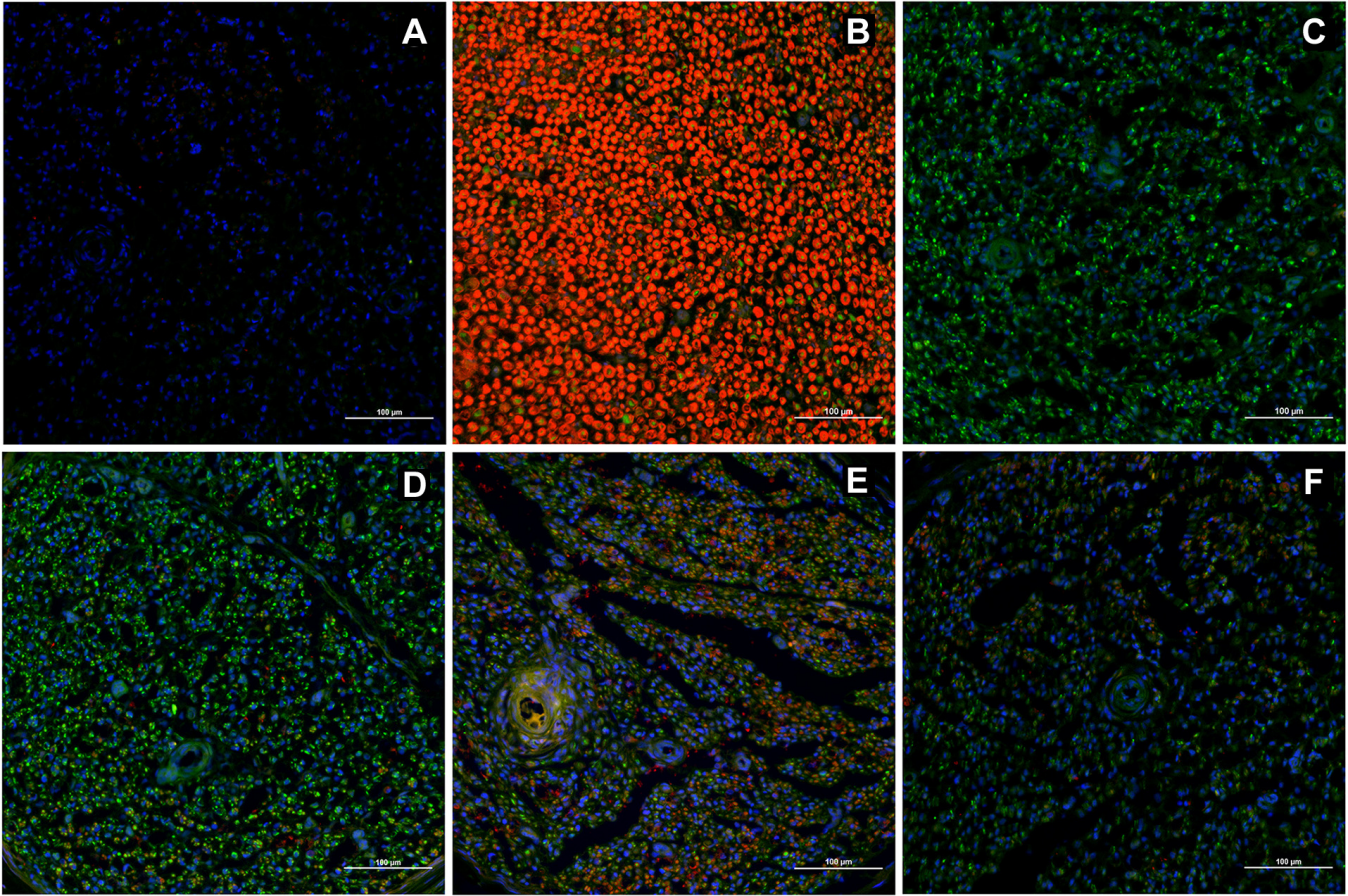
Neurofilament M (NF-M) (axonal marker, green) and myelin (SC marker, red) expression. The images were cropped from images in Fig 4. Scale 100 μm. Images: (A) negative control; (B) naive nerve; (C) control nerve; (D) CH; (E) IGF-1; (F) IGF-1+CH.

Fig 5 shows the green channel (NF-M staining) that was intentionally switched off in Fig 4. Here, NF-M counter-staining is shown together with myelin staining.

**Fig 6:**
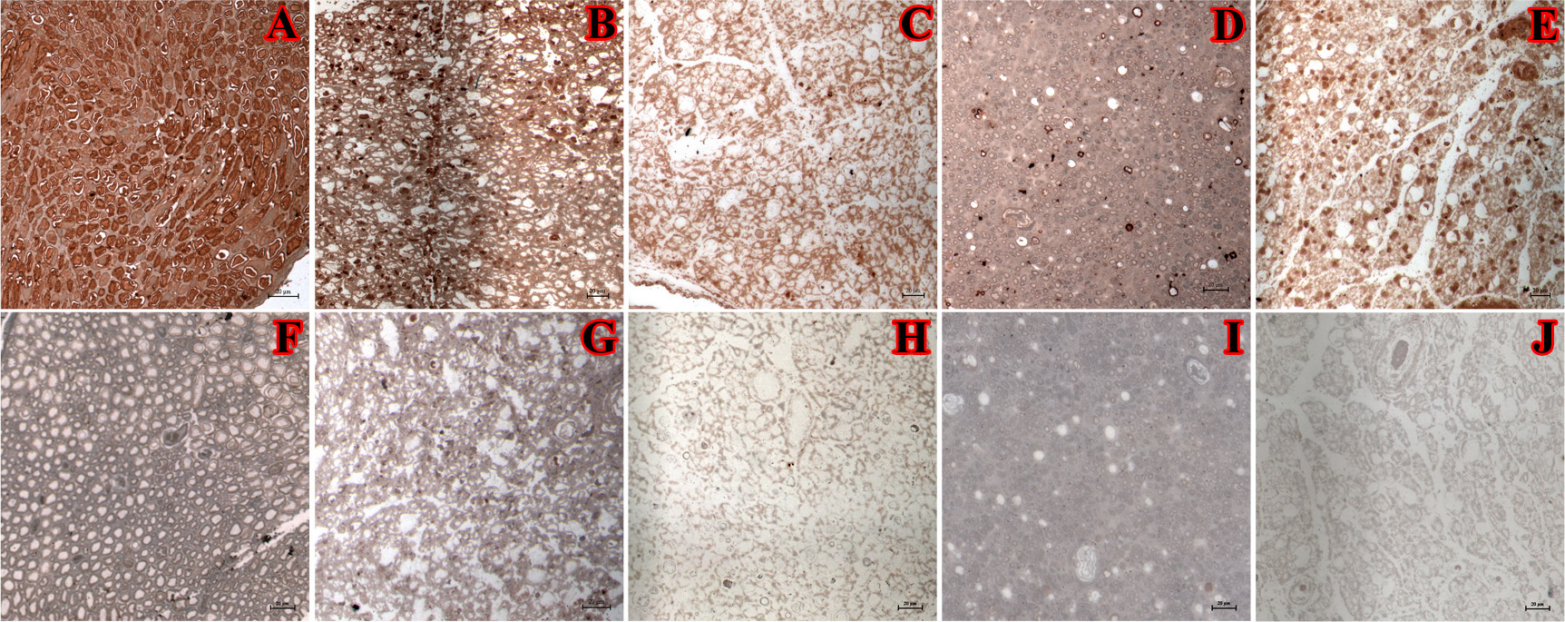
Chondroitin-4-sulphate (proteoglycan) staining in nerves at 5-week end point. Images: (A, F) naive nerve; (B, G) control nerve; (C, H) IGF-1; (D, I) CH; (E, J) IGF-1+CH. Scale is 20μm, magnification 20x. The images F-J show absence of staining in samples without primary antibody staining (Slight variation in background staining in these samples may be due to persistent differences in residual resin remaining in samples).

Anti-chondroitin-4-sulfate antibody was used to evaluate the effect of CH and IGF-1 treatments on presence of growth-restricting proteoglycan at 5 weeks post-surgery (Fig 6). In naïve nerve, the expression of chondroitin-4-sulfate was abundant in axons and SCs. Specifically, most intense staining was observed intracellularly close to membrane area (axon and SC) in comparison to prior studies that report transmembrane and extracellular localization of staining [16]. The expression of this proteoglycan appears to be contact-regulated [17]. Depending on the treatment, proteoglycan was localized to axons (control, IGF-1+CH) or SC (CH group). IGF-1 nerves had negative or only rare axonal staining.

### Histomorphometry of Gastrocnemius Muscle

**Fig 7:**
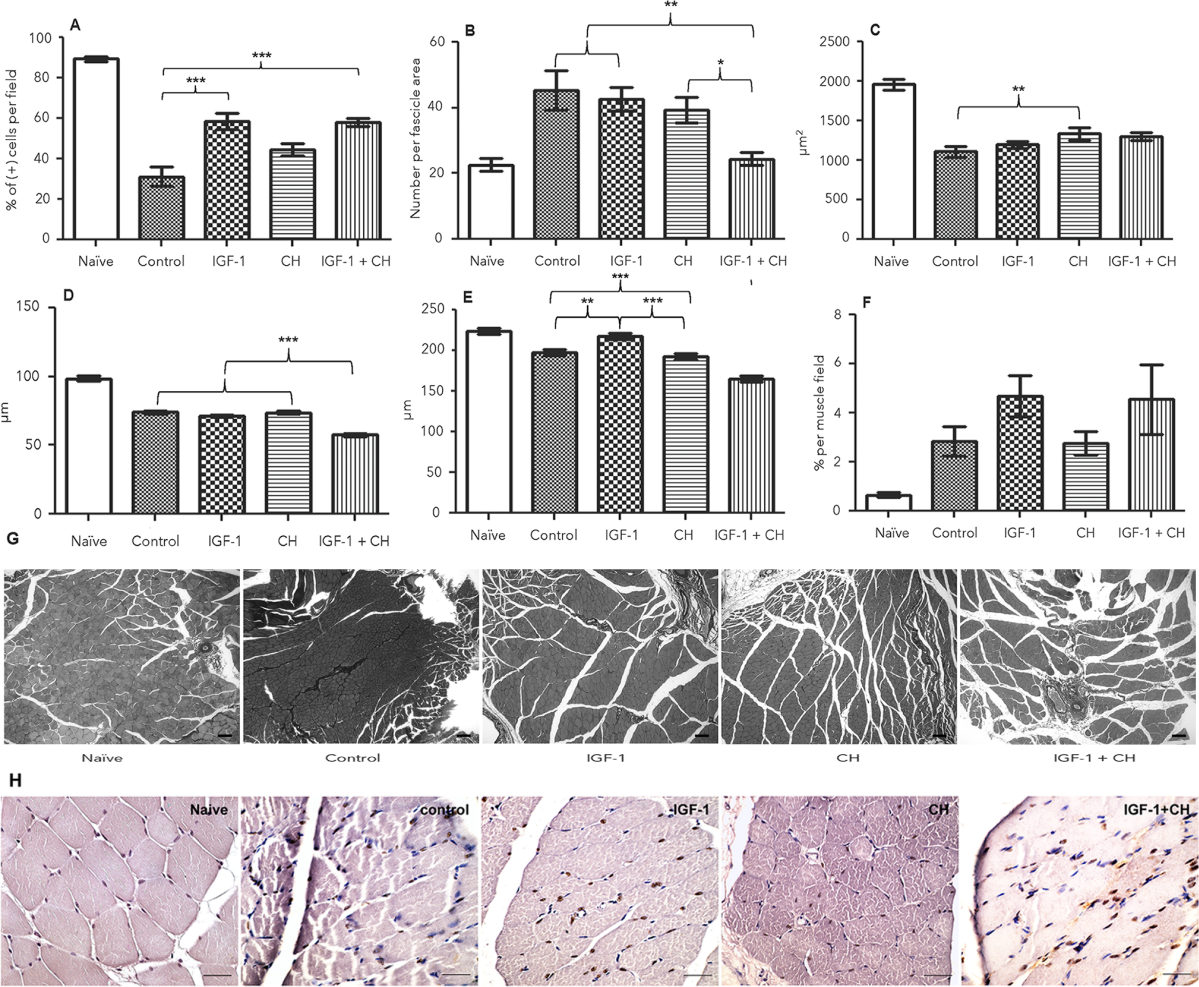
Gastrocnemius muscle histomorphometry. (A) percent of pAkt^Ser437^ nuclei per muscle field (n = 3 rats); (B) number of fibers per muscle fascicle (n = 3); (C) mean muscle fiber size calculated from the fascicular area divided by the number of fibers per fascicle (n = 3); (D) mean perimeter of smallest muscle fiber randomly chosen in the muscle field (n = 3); (E) mean perimeter of largest muscle fiber randomly chosen in the muscle field (n = 3); (F) mean percent of connective tissue per muscle field (n = 3); (G) example of muscles stained with Trichrome, magnification 10x (scale 100 μm); (H) examples of pAkt-positive stained nuclei in muscles, magnification 40x.

Muscle samples were examined with anti-pAktSer473 antibody to determine whether 200 ng of IGF-1 was sufficient for activation of PI3K-Akt signaling in muscles. Phosphorylation of Akt pathway at Ser473 position is known to be a marker for IGF-1 action in the tissue [18]. The number of pAkt-positive stained nuclei increased 1.9 fold in both the IGF-1 and IGF-1+CH groups (mean +/- SEM: 58.44+/-3.996 and 58.05+/-1.826, p = 0.001 and p = 0.002, respectively) compared to controls (mean +/- SEM: 31.29+/-4.827), whereas a moderate increase was seen in the CH group (Fig 7A and 7H). However, the number of positively stained nuclei in IGF-1 treated groups was 1.5-fold less than in naïve muscles (mean +/- SEM: 89.54+/-1.225) [Fig 7A and 7H].

Fascicular hyperplasia of gastrocnemius muscles was observed across all groups except for IGF-1+CH treatment (Fig 7B). Although muscle cross-section area shrank by an average of 1.4-fold by the fifth week after transplantation, the mean fiber size in the CH group was bigger (mean +/- SEM: 1355+/-83.71, p = 0.0213) than in the controls (mean +/- SEM: 1087+/-88.65) (Fig 7C). Normal gastrocnemius muscle contains mixed fiber types: type I (slow oxidative, small size), type IIA (fast oxidative-glycolytic, intermediate size) and type IIB fibers (fast glycolytic, largest). We did not perform type-specific staining, but rather focused our analysis on measuring perimeters of small and large fibers. The mean size of small fibers was similar across groups except for the IGF-1+CH muscles, which had the smallest fibers (Fig 7D). However, large fibers in the IGF group were similar to naïve and significantly bigger than in remaining groups (Fig 7E) (as assessed on Tukey’s, p<0.05, n = 3). Connective tissue content in both IGF-1 and IGF-1+CH treated muscles was increased although with no statistically significant difference (Fig 7F).

## Discussion

Functional recovery after peripheral nerve injury and repair depends on multiple intrinsic and extrinsic factors, which determine neuronal survival after axotomy. This is facilitated by myriad neurotrophins and neuropoietic factors including IGF-1 and CH. IGF-1 is a not only neurotrophic but also myotrophic (induces satellite cell proliferation, differentiation and muscle hypertrophy) and angiogenic (via VEGF) [3, 10–13]. Thus it modulates multiple pathways contributory to optimal neuroregeneration. CH on the other hand enhances nerve regeneration and myelination, attenuates scar formation, and promotes functional nerve recovery. Primarily, CH inhibits chondroitin sulfate proteoglycans (CSPGs), known negative regulators of axonal regeneration after injury. CSPGs promote axonal death and prevent SC access to pro-regenerative laminin. CH degrades the inhibitory CSPGs, thus facilitating axonal regeneration [7–9, 14–16].

Our prior experience in rodent limb transplantation confirms that there is only a finite temporal window exists for making accurate assessments of peripheral (sciatic) nerve regeneration in this model. In prior studies (unpublished data) we found that neurotherapeutic interventions (such as tacrolimus) can cause increases in fiber counts, myelin or SC parameters along with changes in functional outcomes (as seen on Cat Walk or sciatic function index or Swim Test) in this model. Notably, however, when compared to controls (naïve untreated animals with neurorrhaphy) these differences in parameters were significant only up to 40 days. After this time point, there was a “blow-through” effect observed in limb transplanted rats whereby an advancing nerve front overcomes an experimental defect, rendering experimental groups indistinguishable from untreated controls at late time points. In essence, there was a type II error—in which a difference exists between groups but fails to be detected—may be more common than generally recognized. Since the rate of recovery of nerve function was the key research question, and our goal was to preserve key differences in neurotherapeutic efficacy on the nerves, we used a time point less than 40 days in this current study (5 weeks or 35 days).

**Fig 8:**
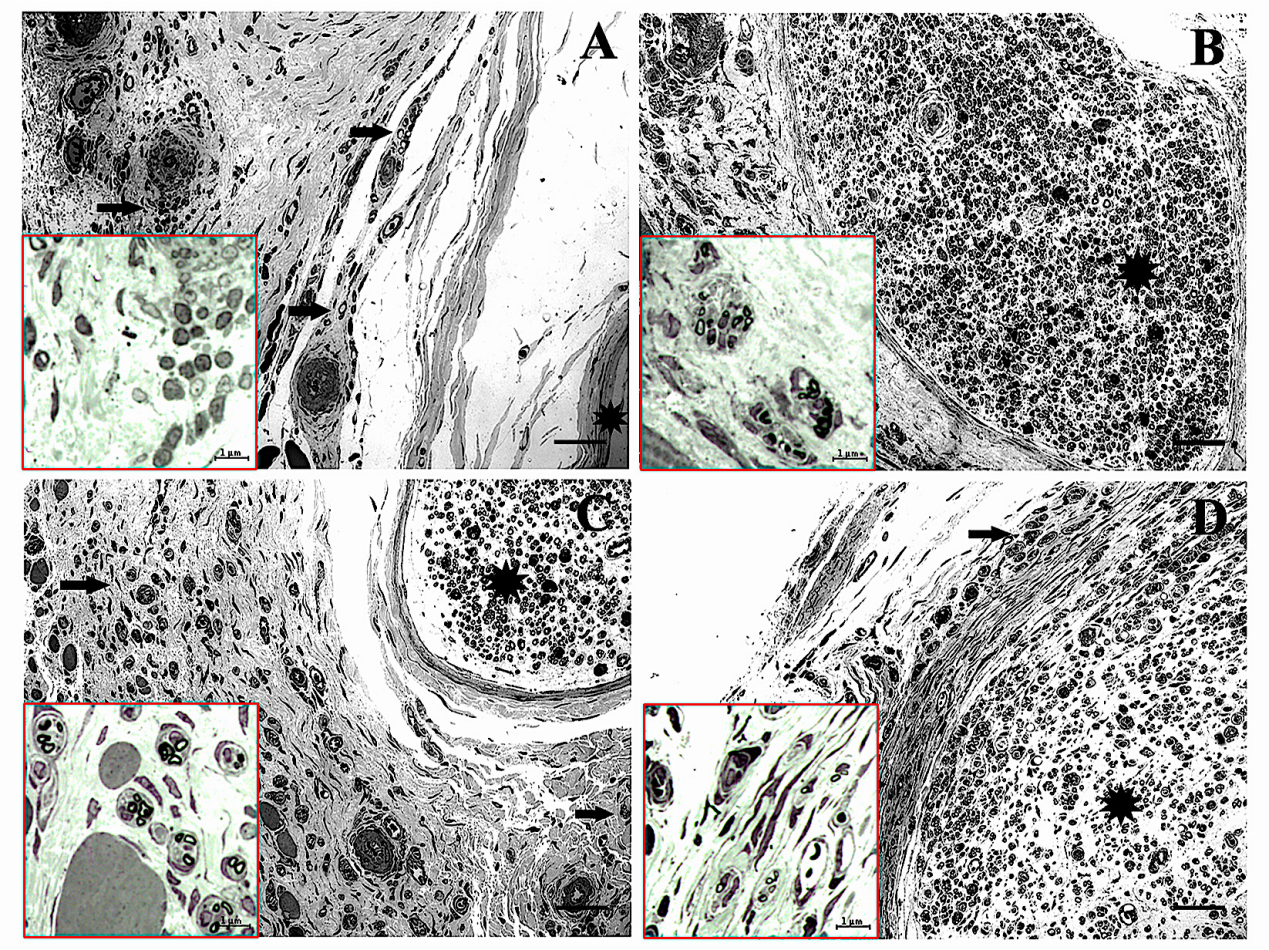
Light-microscopic overview of extrafascicular nerve zones. Toluidine blue stained sections are shown with arrows indicating extrafascicular nerve fibers and stars indicating nerve area. (A) Control sample, (B) CH sample, (C) IGF-1 and (D) IGF-1+CH. Magnification (20x), scale is 50μm. Insets show detail of extrafascicular myelinated axons.

We previously reported the effect of CH monotherapy on nerve regeneration in a similar rat VCA model [14]. The current research differed from our prior study in multiple aspects preventing a head-to-head comparison. Variables included but were not limited to differences in dosing, route of administration, or batch (or vendor) of CH used and the software used in measurements [binary image software (semi-manual) in earlier versus MetaMorph (manual) in current study]. Nevertheless, the findings of Hattori et al. on the effects of CH [same manufacturer and the dose (1U/30μl) as our current study] in a nerve transection and repair model in Sprague-Dawley rats are comparable to our current results with CH monotherapy [15]. One of these findings included reduction in the number of extrafascicular regeneration of axons with CH treatment. Extrafascicular and retrograde regeneration are the main cause of neuropathy, manifesting in loss of, or abnormal nerve function over time [19]. Although, we did not study this process in detail in our samples, some degree of extrafascicular sprouting was seen on light microscopy examination in all groups (least marked in controls, CH and IGF-1 + CH groups and most prominent in the IGF-1 group) (Fig 8).

IGF-1 is chemoattractive for regenerating axons and has been shown to stimulate chemotaxis of neuroblasts [20]. While some extrafascicular fibers resembled degenerated fibers, extrafascicular sprouting in IGF-1 treated nerves was associated with formation of nerve fascicles as shown in Fig 8. Some of the intraneurally injected IGF-1 could have extravasated into surrounding tissues contributing to this phenomenon. Regardless, the effect of IGF-1 therapy on extrafascicular or retrograde nerve regeneration merits further investigation.

Other studies report that IGF-1, unlike CH, induces dose-dependent synthesis of aggregating proteoglycans that are major growth inhibitory components of the glial scar tissue [21–25]. In our study, chondroitin-4-sulfate was present in the axons of IGF-1 and IGF-1+CH nerves but not in CH treated nerves as demonstrated with anti-chondroitin-4-sulfate staining (Fig 6). CH mediates clearance of proteoglycans that block intrafascicular axonal growth by suppressing CSPGs [7–9].

To our knowledge, this is the first report of the use of IGF-1 (alone) or IGF-1+CH (in combination) as neurotherapies in VCA. Our study confirms that IGF-1 and CH monotherapy significantly improves nerve and muscle regeneration. Each neurotherapeutic agent produced a unique profile of quantitative (nerve and muscle histomorphometry) and molecular (IHC) responses. Contrary to our a priori hypothesis, the results of combined therapy were not as effective as IGF-1 or CH monotherapy. Interestingly, we found that the majority of nerve samples from IGF-1+CH group had high levels of hyperplasia. Fascicular area in controls, IGF-1, and CH groups was 53.6%, 40%, and 52.3%, respectively, whereas in the IGF-1+CH group was on average 87% more than naïve nerves (Fig 1C). Total area of myelinated fibers (MF) per nerve cross-section was highest in IGF-1+CH (22%) and IGF-1 (17%) groups, followed by the CH group (13%) and controls (8.5%). Fibers in the IGF-1+CH nerves were larger compared to other groups. The total numbers of MF per nerve was similar in both IGF-1+CH and IGF-1 groups while in naive nerves, MFs occupied approximately 74% of the nerve area. The hyperplasia seen in IGF-1+CH nerves may be due to both increase in MF area and enlarged endoneurium, which includes SCs, fibroblasts, blood vessels, extracellular material, and/or a higher number of non-myelinating axons. Indeed, the increase in cell number per nerve area in the experimental groups was clearly evident (Figs 4 and 5). The number of cells per nerve field was also variable; similar to variations in fascicular area. The IGF-1+CH group had a seven-fold increase in cell number per field as compared to naïve nerves, which was the highest among all groups. In naïve nerves, the majority of cells in the nerve cross-section are SCs, cells crucial to overall nerve regeneration [26]. Therefore, the underlying processes of regeneration can be revealed through characterization of their proliferation and differentiation markers such as GFAP (immature/non-myelinating), Oct6 (pre/pro-myelinating), Krox20 and S100 (mature/myelinating). According to IHC analysis, the majority cells in IGF-1+CH nerve were not SCs. It is likely that they were fibroblasts representing scar tissue. The staining showed signs of poor axonal integrity and poor expression of SC proliferation and differentiation markers. Notably, GFAP expression was barely detectable in both IGF-1+CH and IGF-1 samples. We did not investigate the temporal expression of GFAP in our samples, but we found a report of gradual reduction in GFAP expression following IGF-1 treatment in a rodent model of depression [27]. Our finding may be the first report of down-regulation of GFAP after intraneural injection of IGF-1 and merits further investigation. We used an anti-GAP43 antibody to characterize the extent of regeneration. Similar to GFAP, GAP43 expression was slightly lower in IGF-1 and IGF-1+CH treated groups. Earlier studies using double antibody labeling, found that both proteins are co-expressed in non-myelinating SCs [26]. This may explain lower expression of both proteins in the IGF-1+CH group. We found that the IGF-1 group demonstrated similar to naïve nerves pattern of expression of mature (S100, Krox20) and pre-myelinating (Oct6) SC markers (Fig 2). Moreover, gross myelin expression per cross-section in IGF-1 group was highest among groups, indicating normalization of SC function (Fig 4). It is known that in uncontrolled conditions, GAP43 expression gradually fades as neuromuscular connectivity and innervation is established [28]. A decrease in GAP43 expression in IGF-1 nerves may also be influenced by extrafascicular axonal regrowth into surrounding muscles. We did not evaluate GAP43 expression outside of the nerve cross-section, but this can be addressed in future studies. Contrary to IGF-1 treated nerves, CH nerves demonstrated upregulation of most markers. Although the intensity of Krox20 expression was similar in both groups, the stained cells did not have the same pattern. The staining with NF-M antibody indicated the presence of many maturing axons, but myelination was rather sparse (Figs 2 and 4), suggesting that the majority of SCs were in the pre-myelinating stage and not in tight junctions with axons (myelination is usually initiated by contact with axolemma) [29]. This could indicate that SCs in CH-treated nerve were still undergoing active regeneration.

It is well documented that muscle recovery or protection from atrophy depends on timely and adequate nerve regeneration [30]. IGF-1 or CH treatments improved nerve histomorphometry, but these agents differed in their effects on nerve IHC. Similar to total nerve number, the mean muscle area was comparable across experimental groups except for CH group. The largest fibers were seen in the IGF-1 group while smallest fibers were found in the IGF-1+CH group. Further investigation may be needed to evaluate whether there is positive role for the extrafascicular effects of IGF-1 on muscle regeneration.

Taken together, our study investigated whether targeted delivery (to sites of nerve injury or repair) of low dose IGF-1, CH, or their combination in a clinically relevant regimen (single injection during surgery) on a background of low dose FK506 monotherapy was effective in improving nerve regeneration after VCA. Despite improvements in some histomorphometric and immunohistochemical parameters in neuromuscular components, our results did not fully support our a-priori hypothesis that combination therapy with IGF-1 and CH would boost nerve regeneration after VCA. The finding does not completely exclude the synergistic or additive potency of these agents but rather suggests that further fine-tuning of combined neurotherapies may be required. Further insights into the dosing, frequency and timing of IGF-1 and CH are needed to potentially improve and optimize their effects on neural, muscular or vascular regeneration after VCA.

In conclusion, this study sheds preliminary insights on the promising effects of IGF-1 and CH on histomorphometric and immunohistochemical surrogates of neuroregeneration in a rodent VCA model. We limited our study end point to 5 weeks based on the timeline of the “blow through” phenomenon as reported in rodent peripheral nerve regeneration [31]. However, future investigation must focus on longer-term studies in large animal pre-clinical models that address the potential of these novel neurotherapeutic agents on improving neurofunctional outcomes after VCA.

## Supporting Information

S1 Fig (TIF)

S2 Fig (TIF)

S1 Table Functional characteristics of Schwann cell markers (Literature Review). (DOCX)
