# Supplementary figures and images for: IGF-1 and Chondroitinase ABC Augment Nerve Regeneration after Vascularized Composite Limb Allotransplantation
Source: PLoS One. 2016 Jun 7;11(6):e0156149. doi: 10.1371/journal.pone.0156149 (PMC4896437; doi:10.1371/journal.pone.0156149)

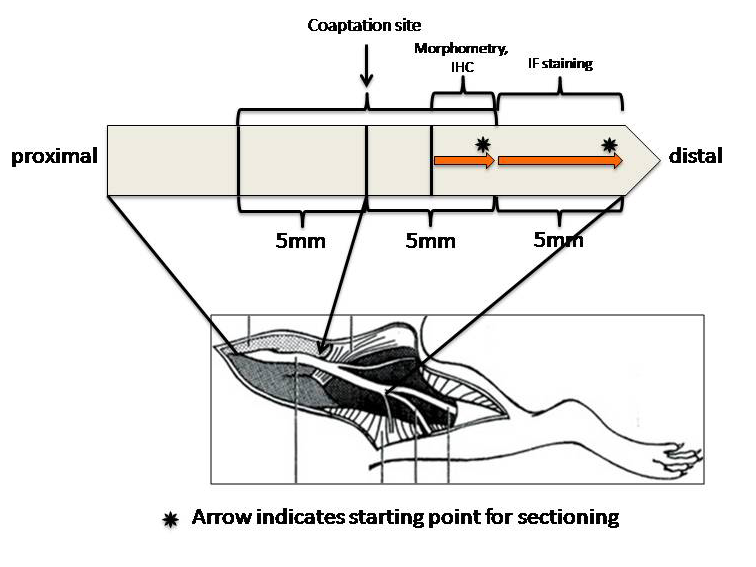

Supplement: S1 Fig — (TIF) [file pone.0156149.s001.tif]

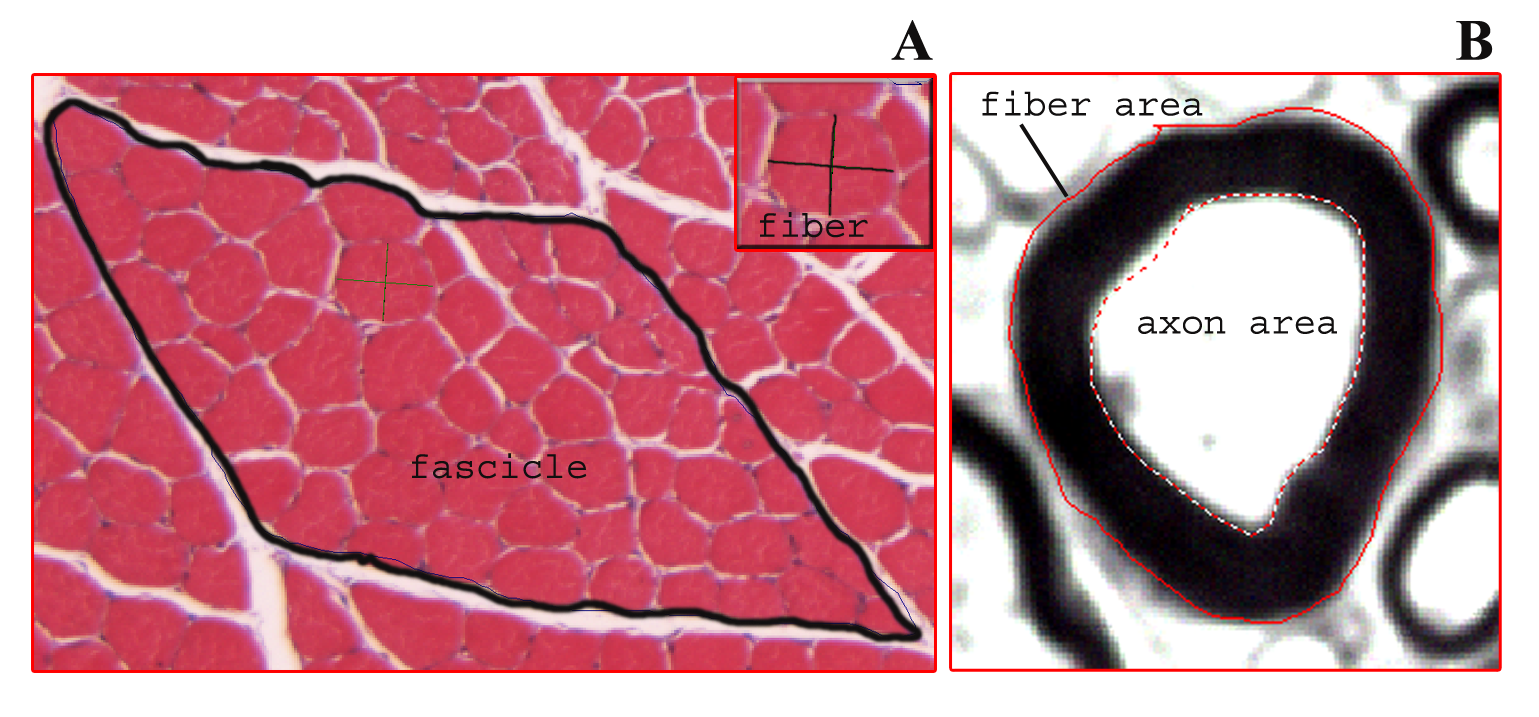

Supplement: S2 Fig — (TIF) [file pone.0156149.s002.tif]
