# Supplementary material for: IGF-1 and Chondroitinase ABC Augment Nerve Regeneration after Vascularized Composite Limb Allotransplantation
Source: PLoS One. 2016 Jun 7;11(6):e0156149. doi: 10.1371/journal.pone.0156149 (PMC4896437; doi:10.1371/journal.pone.0156149)
Supplement: S1 Table — (DOCX) [file pone.0156149.s003.docx]

Table 2 Functional characteristics of Schwann cell markers from literature review.

| Antigen | Marker | Function |
| --- | --- | --- |
| GAP43 | Regeneration, axonal elongation; pathway finding | - Expressed in astrocytes type 1, astrocytes type 2 and oligodendrocytes, mature non-myelinating Schwann cells - Rapidly transported membrane-bound protein - Closely correlates with axonal regeneration and expression of class II tubulin - Participates in structural remodeling and membrane formation, regulate cell shape - Regulated at several levels such as synthesis, transport and degradation - Downregulated by reinnervation - RNA increase up to 10 times at 2d post-trauma, decrease gradually by 37d (our study – 35d) - Schwann cells secrete factors that regulate Gap43 expression |
| S100 | Schwann cell marker | - Commited Schwann cells: non-myelinating and myelinating & peripheral neurons - Regulates cell-cell communication, cell growth, cell structure, energy metabolism, contraction and intracellular signal transduction - Function as intracellular calcium-modulated proteins - If added to culture, promotes neurite extension, can act as extracellular factor - S100 level is upregulated in differentiation and normal development - Implicated in survival/apoptosis, induce apoptosis if overexpressed - Regulate cell shape through involvement of all three components of cytoskeleton: microtubules, actin filaments and intermediate filaments - GFAP is a potential target of S100, S100 binds to GFAP and inhibits its assembly into intermediate filaments - Binds to tubulin and regulates assembly/disassembly of microtubules - Implicated in regulation of energy metabolism because of its interactions with number of enzymes and mobilization of glucose - Increased expression of S100B can result in hyperactivity - Transection of sciatic nerve induce S100 expression in neurons - The expression of S100 in Schwann cells is regulated by Schwann-related cell-axonal contact - S100 marks the transition precursor to Schwann cell phenotype |

| Oct6 (SCIP) | Pre-myelinating Schwann cells, dividing cells | - Level of expression determines fate: increase can lead to differentiation and early myelination and hypermyelination - Lower expression of Oct6 can result in longer time for differentiation program to complete - Regulates Krox20 expression - Myelination is delayed in the absence of Oct-6 - Highly expressed in Schwann cells of regenerating distal nerve stump 8d after axotomy - Differentiation of Schwann cells is arrested at pro-myelin stage in Oct6 and Krox20 null mice, arrest is transient in Oct6 mutants, while arrest is prominent in Krox20 mutant - Depends on continued axonal contact - Oct6 is strongly down-regulated after the pick of myelination |
| --- | --- | --- |
| GFAP | Immature, non-myelinating Schwann cells, proliferation | - GFAP is a glial-specific member of the intermediate filament family - Appears in relatively late stage in Schwann cell development, down-regulated in Schwann cells that form myelin - Expressed in astrocytes - In injury, axonal regeneration was delayed in mutant GFAP mice due to reduced Schwann cell proliferation - Vimentin and nestin probably compensate for the absence of GFAP - GFAP and vimentin link ECM (extracellular matrix) via two distinct pathways |
| Krox20 | Myelinating Schwann cells | - Krox20 expression is maintained through mechanism that does not involve Oct6 - Differentiation of Schwann cells is arrested at pro-myelin stage in Oct6 and Krox20 null mice, but arrest is transient in Oct6 mutants, while arrest is prominent in Krox20 mutant - Regulates cell cycle by reduction in c-Jun protein level, triggering exit from the cell cycle and protection from apoptosis - Krox20 requires continues neuronal signaling via direct axonal contact - Akt activation is essential for Krox20 expression |
| NeurofilamentM NFM) | Axonal marker | - Undergo slow axonal transport, decreased in regenerating axons - Correlates with radial rather than longitudinal growth of axons - Major intrinsic determinant of axonal caliber in myelinated nerve fibers |
